# Design and Initial Characterization of a Small Near-Infrared Fluorescent Calcium Indicator

**DOI:** 10.3389/fcell.2022.880107

**Published:** 2022-06-29

**Authors:** Mikhail E. Matlashov, Jorge Vera, Ludmila A. Kasatkina, Kamran Khodakhah, Vladislav V. Verkhusha

**Affiliations:** ^1^ Department of Genetics and Gruss-Lipper Biophotonics Center, Albert Einstein College of Medicine, Bronx, NY, United States; ^2^ Department of Neuroscience, Albert Einstein College of Medicine, Bronx, NY, United States; ^3^ Medicum, Faculty of Medicine, University of Helsinki, Helsinki, Finland

**Keywords:** GECI, iRFP, FRET, calcium imaging, far-red, biosensor

## Abstract

Near-infrared (NIR) genetically encoded calcium indicators (GECIs) are becoming powerful tools for neuroscience. Because of their spectral characteristics, the use of NIR GECIs helps to avoid signal loss from the absorption by body pigments, light-scattering, and autofluorescence in mammalian tissues. In addition, NIR GECIs do not suffer from cross-excitation artifacts when used with common fluorescent indicators and optogenetics actuators. Although several NIR GECIs have been developed, there is no NIR GECI currently available that would combine the high brightness in cells and photostability with small size and fast response kinetics. Here, we report a small FRET-based NIR fluorescent calcium indicator iGECInano. We characterize iGECInano *in vitro*, in non-neuronal mammalian cells, and primary mouse neurons. iGECInano demonstrates the improvement in the signal-to-noise ratio and response kinetics compared to other NIR GECIs.

## Introduction

Genetically encoded fluorescent calcium indicators (GECIs) engineered from fluorescent proteins (FPs) have long been used in neuroscience for imaging neuronal activities. GECIs allow the optical monitoring of neuronal firing in multiple individual cells and can be genetically targeted to specific subsets of neurons. GECIs based on green fluorescent protein (GFP), including indicators of the GCaMP (Chen et al., 2013; Dana et al., 2019) or Twitch (Thestrup et al., 2014) families are now widely used for studies of brain circuits (Broussard et al., 2014; Lin and Schnitzer, 2016; Luo et al., 2018). However, GFP-based GECIs have several drawbacks that limit their use. They require illumination with blue–green light (480–520 nm), which is strongly absorbed by body pigments and highly scattered in brain tissues. Therefore, neural imaging in deep layers requires invasive surgical procedures, such as implanting optical fibers or prisms (Jennings et al., 2015; Gaffield et al., 2016). In addition, the excitation spectrum of such indicators overlaps with the activation spectrum of the most widely used genetically encoded channelrhodopsin actuators, such as channelrhodopsin ChR2 (Boyden et al., 2005). To overcome these drawbacks, several red-shifted GECIs have been developed using red FPs, such as RGECO (Zhao et al., 2011) or RCaMP (Akerboom et al., 2013; Inoue et al., 2015). However, these indicators have not completely solved the problem of high light absorption by brain tissues and require the careful adjustment of light intensities to be combined with channelrhodopsins.

The development of near-infrared (NIR) FPs of the IFP (Shu et al., 2009; Yu et al., 2015) and iRFP (Shcherbakova et al., 2016; Matlashov et al., 2020) families allowed to shift fluorescence excitation and emission to a near-infrared tissue transparency optical window (650–900 nm). Although these FPs require the binding of biliverdin IXa (BV) linear tetrapyrrole as a chromophore, they are suitable for imaging in mammalian tissues where BV is produced as a result of heme catabolism. So far, only a very few NIR GECIs have been developed (Shcherbakova, 2021), with current state-of-the-art indicators being iGECI (Shemetov et al., 2021) and NIR-GECO2G (Qian et al., 2020). iGECI is a Förster resonance energy transfer (FRET)-based NIR indicator. It has relatively high effective brightness and photostability in mammalian cells and allows imaging in deep layers of the mouse cortex. However, iGECI suffers from slow signal decay (t_off_) and has a large molecular weight of 86 kDa, which may affect protein targeting and packaging efficiency in adeno-associated virus (AAV). NIR-GECO2G is an intensiometric indicator composed of a single modified mIFP protein. It is characterized by fast on and off kinetics and a high magnitude of response per single action potential (up to 17%) in cultured neurons (Qian et al., 2020). This indicator, however, suffers from relatively low brightness in mammalian cells and fast photobleaching.

Both iGECI and NIR-GECO2G can be used in a spectral crosstalk-free combination with popular GFP-based indicators and channelrhodopsin actuators, allowing the simultaneous monitoring of several cellular parameters or optical modulation and monitoring of neuronal activities. While both indicators have their advantages, they are still far from an ideal NIR GECI, which would combine the high brightness in cells and photostability with fast kinetics and low molecular weight.

Here, we develop a small NIR GECI of 58 kDa based on a FRET pair consisting of miRFP670nano (Oliinyk et al., 2019) and miRFP720 (Shcherbakova et al., 2018), NIR FPs, and a minimal troponin C (TnC)-based Ca-sensitive domain. We describe the molecular evolution and high throughput screening of calcium indicators in bacterial and mammalian cells. We characterize the obtained NIR GECI *in vitro*, in human HeLa cells and mouse primary neurons and compare its performance with iGECI and NIR-GECO2G. We demonstrate that the resultant NIR GECI combines the advantages of all current NIR GECI indicators.

## Results

### Engineering and Molecular Evolution of Small Near-Infrared Genetically Encoded Calcium Indicator Variants

To design iGECInano, we used a Ca^2+^-sensing domain from Twitch-2B (Thestrup et al., 2014) developed from a minimal Ca^2+^-binding domain of troponin C (TnC_m_). Unlike Ca^2+^-sensing domains in iGECI and NIR-GECO2G that are based on calmodulin, TnC_m_ does not require a binding partner peptide and only has two Ca^2+^-binding motifs, called EF-hands. Therefore, indicators with TnC_m_ should have simpler and likely faster conformational changes associated with Ca^2+^-binding and lower calcium buffering in cells. To build a FRET pair, we chose miRFP720 FP as the FRET-acceptor and miRFP670nano FP (Oliinyk et al., 2019) as the FRET-donor. miRFP670nano is a single domain NIR FP characterized by fast maturation time and a twice smaller size compared to all other currently available NIR FPs. To generate an initial library of NIR GECI variants, we fused miRFP670nano and miRFP720 to TnC_m_ directly or *via* randomized linkers of 1–4 amino acid residues in length.

**FIGURE 1:**
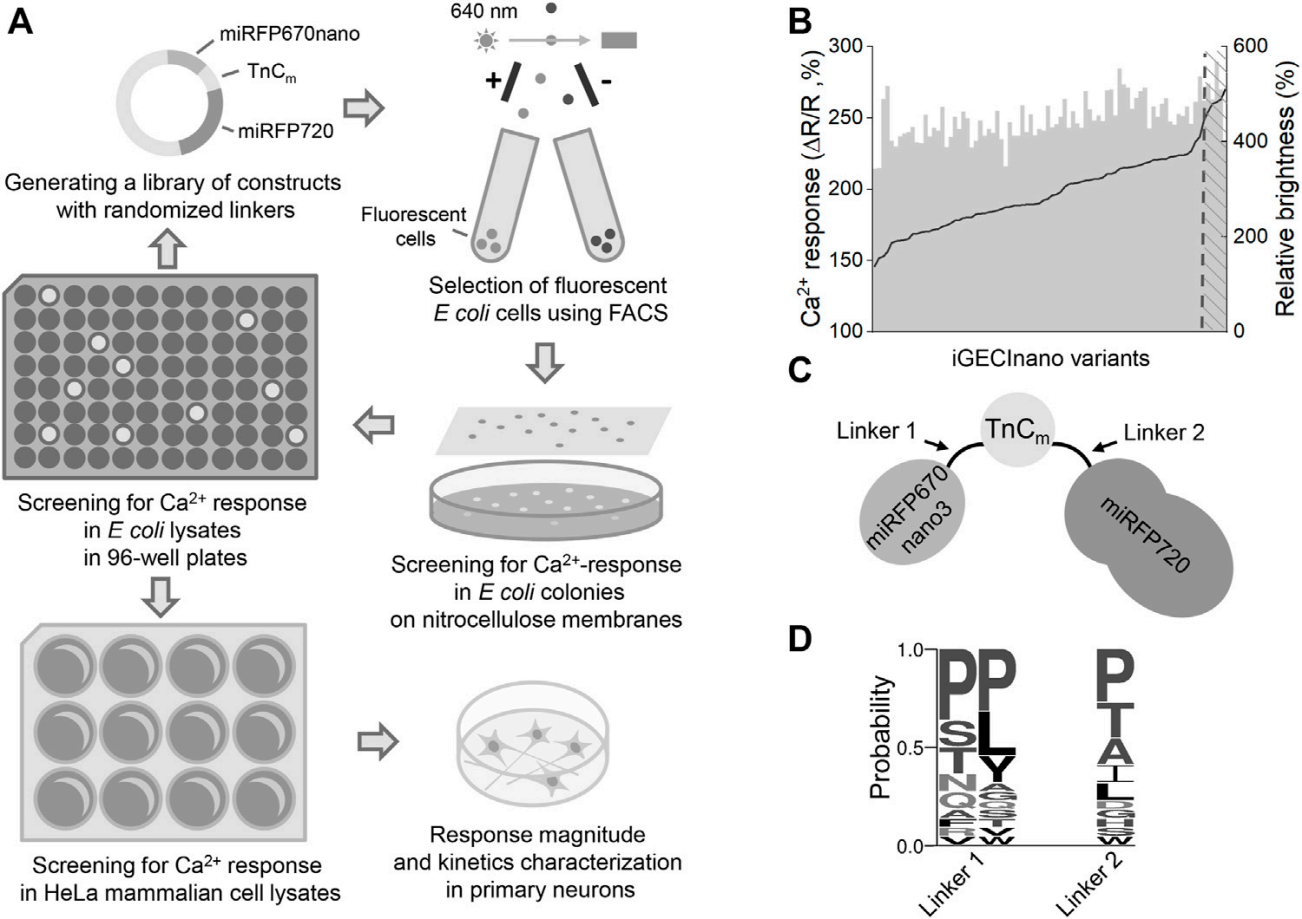
The indicator design and screening strategy. **(A)** The library of the NIR GECI variants with randomized linkers was pre-sorted for positive fluorescence of miRFP670nano and miRFP720. Collected cells were plated on Petri dishes, mature colonies transferred to nitrocellulose membranes and screened for the calcium response by spraying with ionomycin/EGTA solution, then with the Ca^2+^ solution and comparing with the miRFP670nano/FRET ratio. The colonies demonstrating the highest response were further used for screening in bacterial lysates, then in mammalian lysates. The final indicator variant was tested in cultured neurons. **(B)** Evolution of iGECInano variants in bacteria. The amplitude of the FRET/donor ratio change in response to 1 mM Ca^2+^ (black) and relative brightness (gray) of the tested indicator variants. The brightness of the iGECInano variants was normalized on the brightness of iGECI. Variants with a response above the 250% threshold were sequenced and tested in HeLa cells. **(C)** Schematic of the iGECInano structural design. A minimal TnC-based Ca^2+^-sensing domain TnC_m_ derived from Twitch-2B indicator is sandwiched between a FRET pair of miRFP670nano (FRET donor) and miRFP720 (FRET acceptor). **(D)** The frequency of amino acid residues in randomized linkers of iGECInano variants, having a Ca^2+^ response above 250% threshold.

An initial screening of NIR GECI variants was performed similarly to the process previously described (Shemetov et al., 2021) (Figure 1A). In short, we transformed the library of NIR iGECI variants into BL21 (DE3) bacterial strain expressing heme oxygenase, collected the brightest cells using a fluorescence-activated cell sorter (FACS), plated them on Petri dishes, and then transferred the mature colonies onto a nitrocellulose membrane. The colonies were further screened for functional response by measuring the baseline FRET in the presence of 1 mM EGTA and 50 μg/ml ionomycin, then, they were sprayed with a solution containing 100 mM CaCl_2_ to measure the FRET change in response to Ca^2+^. The NIR GECI variants that had miRFP670nano at the N-terminus and miRFP720 at the C-terminus demonstrated a higher Ca^2+^-response compared to those with the reversed orientation of the NIR FPs. We selected the NIR GECI clones demonstrating the highest Ca^2+^-response for further characterization in bacterial lysates using multiwell plates.

In bacterial lysates, we recorded the fluorescence spectra in the presence of 1 mM Ca^2+^ or 1 mM EGTA. We identified several NIR GECI variants demonstrating an approximately 60% drop of the donor/FRET ratio in response to 1 mM Ca^2+^ (or an approximately 2.5-fold higher donor/FRET ratio in the Ca^2+^-free state compared to Ca^2+^-loaded states). Bacterial lysates of these variants had about 4–6 times brighter fluorescence in the donor channel compared to the lysates expressing iGECI control (Figure 1B), likely due to a higher protein expression level due to smaller size.

NIR GECI variants exhibiting the highest brightness and largest Ca^2+^ response were further tested in lysates of mammalian cells. We transfected HeLa with plasmid-encoding NIR GECI variants under the CMV promoter. 24 h after transfection, 5 μM BV was added, and 48 h after transfection, cells were lysed and their fluorescence spectra were recorded in the presence of 1 mM Ca^2+^ or 1 mM EGTA. The brightest variant with the largest Ca^2+^ response had a linker between miRFP670nano and TnC_m_ consisting of two Pro amino acid residues (L1) and a linker between TnC_m_ and miRFP720 consisting of a single Pro residue (L2) (Figures 1C,D). We named this variant iGECInano. We concluded that, short rigid linkers between NIR FPs and the Ca^2+^-sensing domain were essential for advanced iGECInano properties.

### *In Vitro* Characterization of iGECInano

**FIGURE 2:**
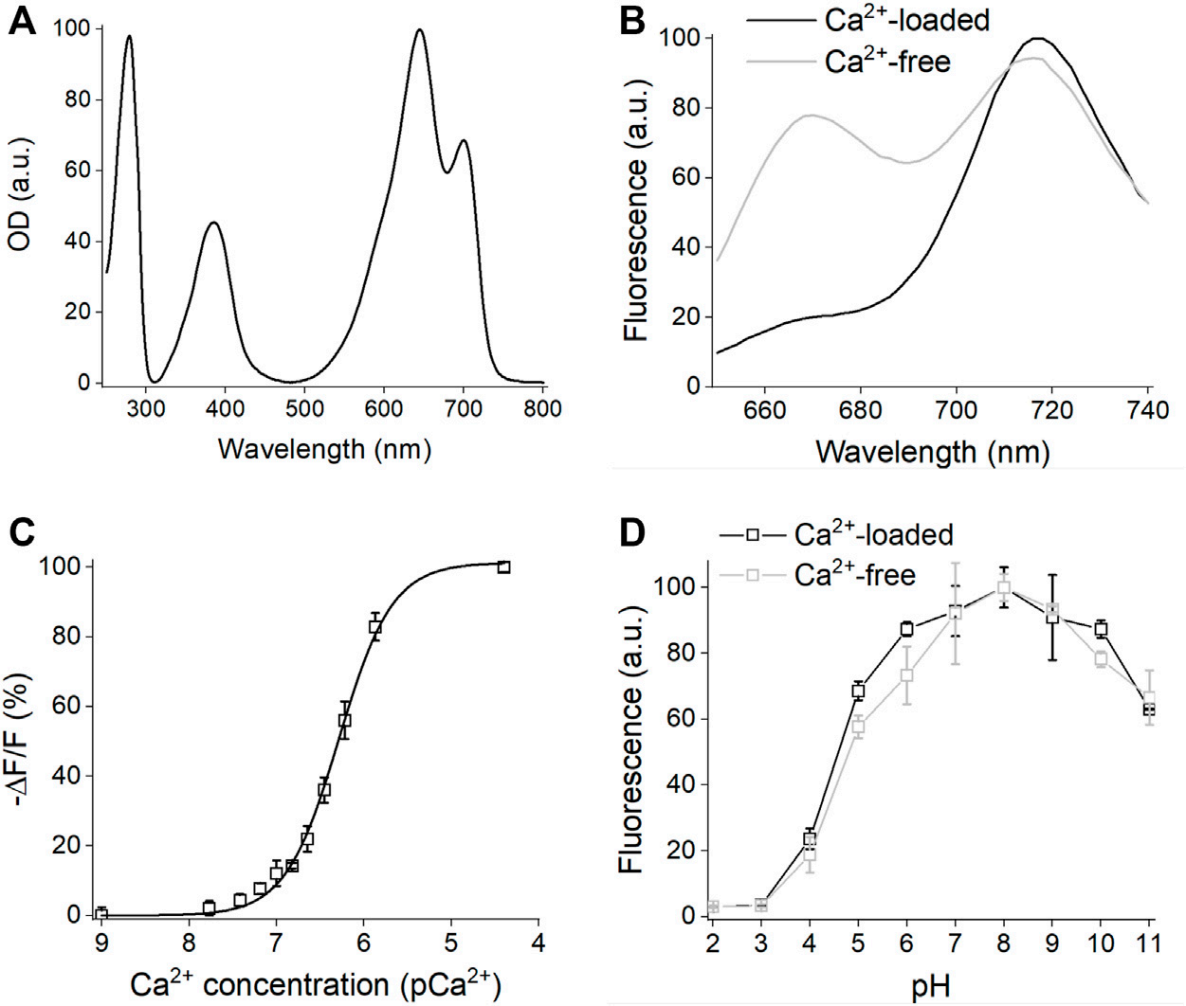
*In vitro* characterization of iGECInano. **(A)** Absorbance spectrum of purified iGECInano. **(B)** Fluorescence spectra of iGECInano in HeLa cells lysate in the presence of 1 mM Ca^2+^ (black line) or 1 mM EGTA (gray line). **(C)** Dependence of purified iGECInano fluorescence on Ca^2+^ concentration. **(D)** Dependence of purified iGECI fluorescence on the pH value in the presence of 1 mM EGTA (gray), or 1 mM Ca^2+^ (black). Error bars are SEM, *n* = 4 experiments.

The absorption spectrum of iGECInano had two major absorption maxima at 640 and 700 nm corresponding to the absorption of miRFP670nano donor and miRFP720 acceptor (Figure 2A; Table 1). iGECInano also had a smaller absorption peak at 390 nm corresponding to the Soret band of BV in NIR FPs, attributed to the absorbance of individual pyrrole rings, and a peak at 280 nm attributed to a sum of absorbances of BV and aromatic residues of the apoprotein. The indicator had very low absorption at 450–500 nm, allowing it to combine with GFP-based indicators and opsin-based optogenetic actuators.

**TABLE 1:** Comparison of iGECInano with other NIR GECIs and widely used GCaMP6s.

GECI name	Ex/Em (nm)	Molecular brightness vs. EGFP, %^a^		p*K*_a apo,_ p*K*_a sat,_	Photostability t_0.5_, s^b^		Effective brightness, %^c^		Dynamic range (ΔF/F), fold	Hill coefficients n_1_ and n_2_	K_d1_ and K_d2_, nM	Rise time, s^d^	Decay time, s^d^	ΔF/F, % per 1 AP		References
		w/o Ca^2+^	with Ca^2+^		HeLa cells	Mouse neurons	w/o BV	with BV						In neurons	*In vivo*	
iGECInano	donor 645/670 acceptor 702/720	n.a.	n.a.	4.6, 4.8	780	n.a.	16	140	4×	1.53	530	0.7	2.4	−21.7	n.a.	This work
iGECI	donor 640/670 acceptor 702/720	37	6.0	4.5, 4.5	1795	1735	46	100	6×	2.5 and 0.90	15 and 890	0.7	14	−5.7 (−12.9 with BV)	∼−5 to 20	Shemetov et al. (2021)
												0.6	5.7	−17.8	n.a.	This work
NIR-GECO1	678/704	12	1.0	5.1, 4.9	100^b^	134^b^	2.4^f^	20^f^	8×	0.99	215 and 885	1.5^f^	4.0^f^	∼−4.5 (∼−10 with BV^f^)	n.a.	Qian et al. (2019)
NIR-GECO2G	678/704	13	1.3	5.3, 4.8	n.a.	∼130^e^	∼5^e^	n.a.	n.a.	0.78	480	∼1.2	∼3	∼−16	n.a.	Qian et al. (2020)
							22 ^(this work)^	75 ^(this work)^				1.8	4.9	−44.9	n.a.	This work
NIR-GECO2	678/704	12	0.75	5.3, 4.8	n.a.	∼130^e^	∼2^e^	n.a.	n.a.	0.94	331	∼1.3	∼3.5	∼−17	n.a.	Qian et al. (2020)
GCaMP6s	497/515	∼5	123	9.8, 6.0	n.a.	n.a.	n.a.	n.a.	60×	2.9	144	0.48	1.8	28	23	Chen et al. (2013)

a Molecular brightness is determined by extinction coefficient multiplied by quantum yield.

b Determined in (Shemetov et al., 2021), using 605/30 nm excitation and 647 nm long-pass emission filters at 14 mW/cm2 light power density at the back aperture of the lens, and normalized to absorption efficiency of the biosensors at 605 nm.

c Determined in transiently transfected live HeLa cells. Effective (a.k.a. cellular) brightness of iGECI with 25 μM of exogenous BV was assumed to be 100%.

d Determined for 1–10 electrical pulses in cultured neurons, without adding exogenous BV.

e Estimated based on data in (Shemetov et al., 2021) and (Qian et al., 2020).

f Measured in (Shemetov et al., 2021). Other NIR-GECO1 data are from (Qian et al., 2019). n.a., not available.

Next, we measured the fluorescence emission of iGECInano in the presence of 1 mM CaCl_2_ (Ca^2+^-loaded state) or 1 mM EGTA (Ca^2+^-free state) in the lysates of HeLa cells. iGECInano fluorescence spectrum had two peaks at 670 and 718 nm, corresponding to miRFP670nano and miRFP720, respectively (Figure 2B). iGECInano exhibited an approximately 400% change in the FRET-acceptor to FRET-donor fluorescence ratio between the Ca^2+^-free and Ca^2+^-loaded states, mainly attributed to the decrease in miRFP670nano3 fluorescence. A small increase of FRET acceptor fluorescence in response to Ca^2+^ likely resulted from the compensation of the decrease of the donor and increase of the acceptor fluorescence signals.

We then measured the Ca^2+^ affinity of iGECInano (Figure 2C). The obtained Kd was 530 ± 20 nM, with a Hill coefficient of 1.53 ± 0.08. As expected, since TnC_m_ has only two EF-hands, it demonstrated the relatively simple dependency of the signal on Ca^2+^ concentration compared to iGECI (Shemetov et al., 2021). Interestingly, the Ca^2+^ affinity of iGECInano was somewhat lower than reported for Twitch-2B (200 nM), which contained the same TnC_m_ domain.

We also studied the pH stability of iGECInano in the presence of 1 mM CaCl_2_ or 1 mM EGTA (Figure 2D). We observed minimal fluorescence changes between pH 6.0–10.0 and a decrease of fluorescence in the acidic range with pK_a_ 4.6 ± 0.1 for Ca^2+^-loaded and 4.8 ± 0.2 for Ca^2+^-free states. This pH dependence was similar to iGECI (Shemetov et al., 2021) and substantially broader than observed for the NIR–GECO series of indicators (Qian et al., 2019).

### Performance of iGECInano in Non-Neuronal Mammalian Cells

**FIGURE 3:**
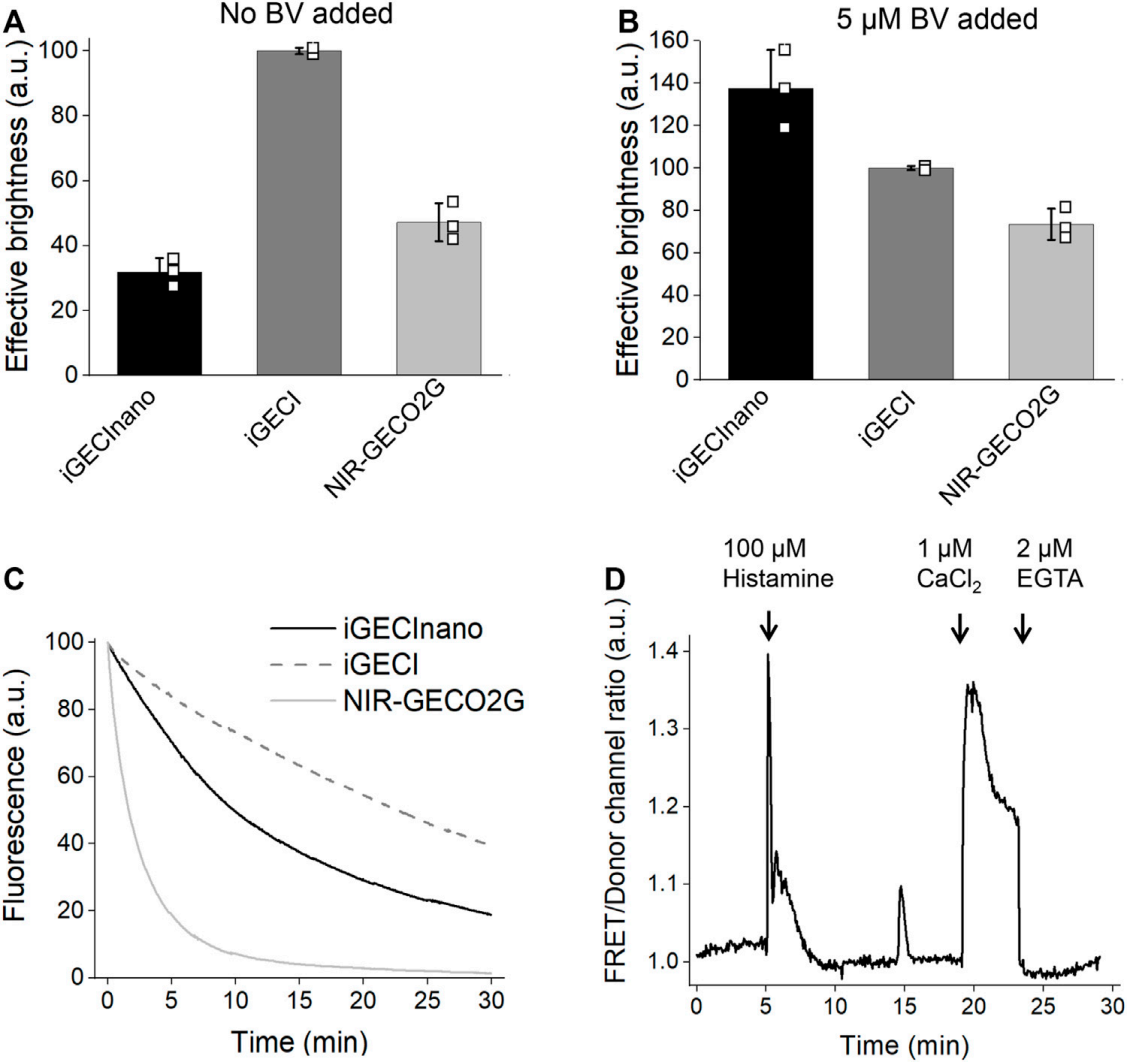
Characterization of iGECInano in live human HeLa cells. **(A,B)** Comparison of iGECInano, iGECI, and NIR-GECO2G brightness in live HeLa cells in the absence of BV **(A)** and in the presence of 5 μM BV **(B)** measured using flow cytometry and normalized on iGECI fluorescence. The 640 nm laser was used for excitation, and a 647 nm long-pass edge filter to detect fluorescence. Fluorescence intensities were normalized to the absorption efficiencies of the indicators at 640 nm. In **(A,B)**, *n* = 3 individual experiments. **(C)** Photobleaching curves of iGECInano, iGECI, and NIR-GECO2G in live HeLa cells excited using a 605/30 nm bandpass and imaged using a 647 nm long-pass filter. Photobleaching data were normalized to the absorption efficiencies of indicators at 605 nm. 5 μM exogenous BV was supplied 24 h before the experiment. *n* = 5 cells. **(D)** Typical Ca^2+^ transients reported by iGECInano in live HeLa cells. Ratio changes of the FRET acceptor (ex. 605 nm, em. 725/40 nm) to the donor (ex. 605 nm, em. 680/20 nm) fluorescence intensities upon treatment with 100 μM of histamine, followed by changing the media to one containing 10 μM ionomycin and 1 mM Ca^2+^ and then 2 mM EGTA.

Next, we compared the effective (cellular) brightness of iGECInano, iGECI, and NIR-GECO2G expressed in transiently transfected HeLa cells under the same CMV promoter. In the absence of an externally added BV, iGECInano was approximately 3-folds dimmer compared to iGECI (Figure 3A). However, in the presence of 5 μM external BV, iGECInano demonstrated about 40% higher brightness compared to iGECI (although not statistically significant, *p* > 0.05) and about 100% higher brightness compared to NIR-GECO2G (Figure 3B). iGECInano had lower than iGECI effective brightness in the absence of exogenous BV, even though neither miRFP670nano nor miRFP720 required BV supply to brightly fluoresce in mammalian cells.

miRFP670nano (Oliinyk et al., 2019) was reported to be more photostable compared to miRFP670 used in iGECI. However, iGECInano appeared to be somewhat less photostable (photobleaching t_0.5_ = 780 ± 60 s) compared to iGECI (t_0.5_ = 1750 ± 150 s). Still, it was significantly more photostable than NIR-GECO2G (t_0.5_ = 75 ± 15 s) (Figure 3C).

Finally, iGECInano allowed reliable recording of histamine-induced Ca^2+^-oscillations in HeLa cells (Figure 3D). Overall, although iGECInano had a relatively low cellular brightness without exogenous BV, its higher photostability and pH dependence allows it to be competitive with NIR-GECO2G. In experiments on cultured cells or tissues, external BV can be easily supplied to increase the iGECInano signal.

### Characterization of iGECInano in Primary Mouse Neurons

**FIGURE 4:**
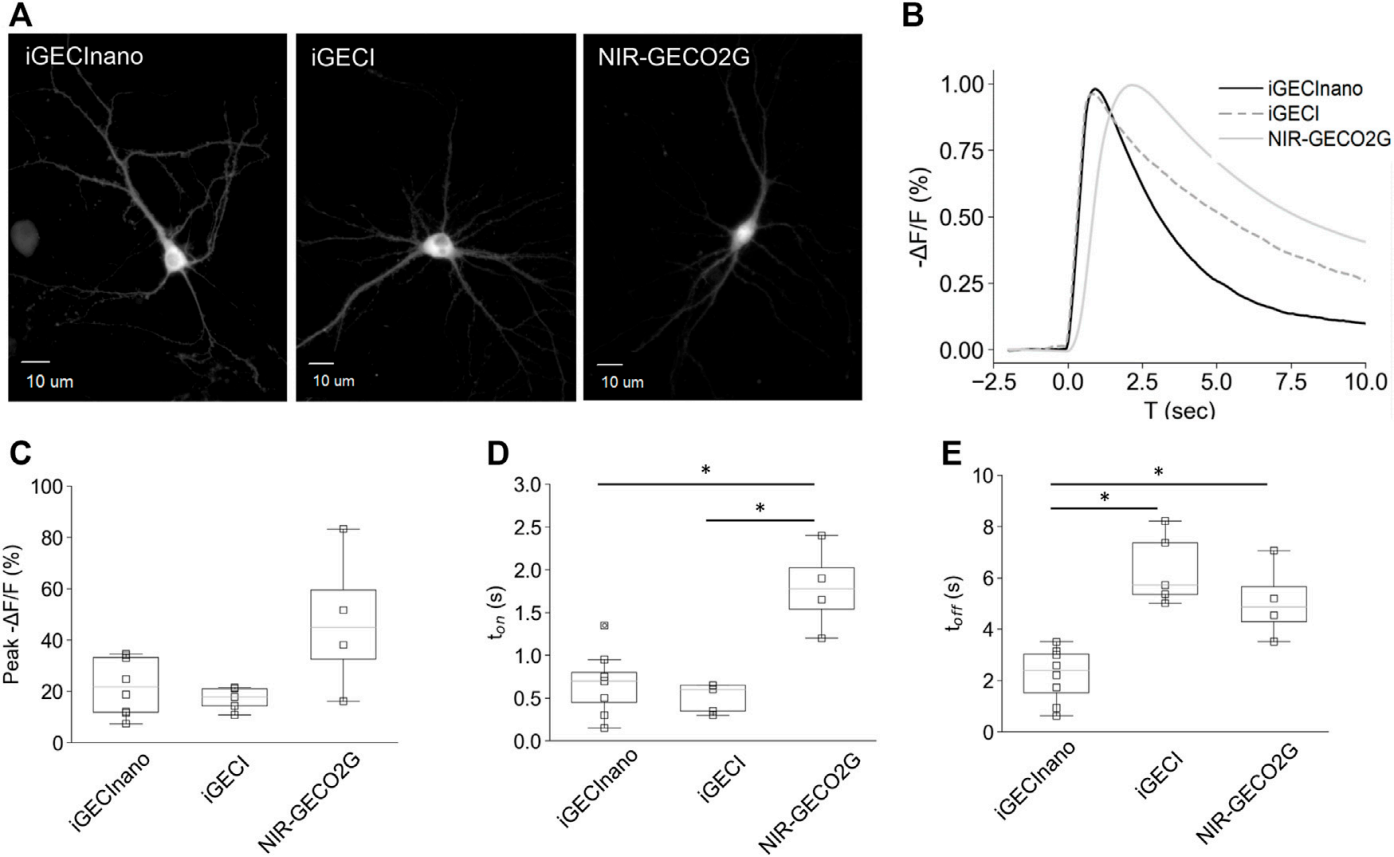
Performance of iGECInano in cultured primary mouse neurons. **(A)** Images of neurons expressing NES-iGECInano, NES-iGECI, and NIR-GECO2G. **(B)** Normalized fluorescence change recorded from cells expressing iGECInano, iGECI, and NIR-GECO2G evoked with a single pulse of electric stimulation (traces represent the mean value). **(C–E)** Box plots showing peak fluorescent **(C)**, time to peak (t_on_) **(D)**, and decay time (t_off_) of the evoked responses **(B)**. Box plots show median, 25 and 75 quartiles, and max and min values. Statistical significance (*p* < 0.05, Kruskal–Wallis and Dunn’s multiple comparison test) is shown with an asterisk (*). Cells per condition: iGECInano (*n* = 8), iGECI (*n* = 5), NIR-GECO2G (*n* = 4).

Next, we compared the performance of NES-iGECInano and NES-iGECI (versions of the indicators fused with a nuclear export signal, therefore localized primarily in the cytoplasm and not in the nucleus), as well as NIR-GECO2G, in mouse primary neurons isolated from the cortex. On seventh DIV, the cultured neurons were transduced with AAVs encoding iGECInano, iGECI, or NIR-GECO2G under the CAG promoter and incubated for 7–9 days in the presence of 5 μM BV. All three indicators demonstrated a uniformly distributed fluorescence in the cytoplasm of neurons (Figure 4A). We then stimulated neuronal activity using 1-ms single pulse electric field stimulation while imaging the fluorescence of cell bodies (Figure 4B). The changes in fluorescence induced after electric stimulation showed a peak between −21.7% and −44.9% among cells expressing different sensors, without a significant difference between groups [mean −ΔF/F of 21.7% (11.8%–33.5%), 17.8% (14.3%–21.1%), and 44.9% (27.1%–67.5%) for iGECInano, iGECI, and NIR-GECO2G; *p* > 0.05 for all paired comparison] (Figure 4C). Values are the mean and (25%–75%) quartiles. To assess the kinetics of the evoked signals in cortical neurons, we first measured the onset of the fluorescence responses. Cells expressing iGECInano and iGECI showed similar time-to-peak values [0.7 s (0.4–0.85 s) vs. 0.6 s (0.35–0.65 s) for iGECInano and iGECI, respectively; *p* = 0.327], while cells expressing NIR-GECO2G presented a response onset of 1.8 s ([1.4–2.2 s), showing a slower kinetics compared to both, iGECInano (*p* = 0.029) and iGECI (*p* = 0.018) (Figure 4D). We then measured the decay of the evoked changes in fluorescence, finding that cells expressing iGECInano showed the fastest kinetics, being ∼2.3 times faster than cells expressing iGECI [2.4 s (1.3–3.1 s) vs*.* 5.7 s (5.4–7.4 s) for iGECInano and iGECI, respectively; *p* = 0.002], and ∼2 times faster than cells expressing NIR-GECO2G [2.4 s (1.3–3.1 s) vs. 4.9 s (4.0–6.1 s) for iGECInano and NIR-GECO2G, respectively; *p* = 0.035] (Figure 4E). Taken together, these data show that iGECInano displays faster kinetics when compared to iGECI and NIR-GECO2G.

The observed faster kinetics probably resulted from a smaller and simpler Ca^2+^-sensing domain of iGECInano, which did not require interdomain interactions like the binding of M13 or RS20 peptides in calmodulin-based indicators. Therefore, iGECInano combines fast response kinetics with favorable photochemical characteristics (Table 1).

## Discussion

Using a rational design followed by random mutagenesis and screening in both bacteria and mammalian cells, we have developed the smallest NIR FRET-based indicator for Ca^2+^ ions, iGECInano. The iGECInano indicator is based on a pair of monomeric NIR FPs engineered from the bacterial phytochromes such as miRFP670nano and miRFP720 and is characterized by high cellular brightness in the presence of BV and fast Ca^2+^-response kinetics (Figures 3, 4). In addition, iGECInano is approximately 1.5 times smaller compared to iGECI (1,563 bp for iGECInano versus 2,394 bp for iGECI) making it suitable for packaging into AAV vectors, which have a strict 4.5–4.9 kbp limit for inserts, including a promoter and other regulatory elements (Dong et al., 1996).

In the presence of exogenous BV, iGECInano was substantially brighter in cells compared to iGECI and NIR-GECO2G. Also, compared to NIR-GECO2G, both iGECI and iGECInano exhibited a broader pH stability and photostability. Surprisingly, iGECInano seems to have a lower affinity to BV as compared to iGECI. Since the miRFP670nano and miRFP720 proteins themselves show bright fluorescence in the absence of external BV when individually expressed in mammalian cells, we think that the current configuration of the indicator may somehow affect BV binding by the NIR FP pair. An additional cycle of evolution of iGECInano in mammalian cells may resolve the problem in the future. For *in vitro* experiments, the fluorescent signal of iGECInano can be enhanced with exogenous BV. At the same time, iGECInano may become a template for further evolution and generation of the first NIR GECI transgenic mouse enabling an efficient incorporation of endogenous BV during protein maturation.

Both iGECI and iGECInano possessed two emission peaks at 670 and 718 nm (Figure 2); however, Ca^2+^-response was mostly characterized by a drop of fluorescence in the donor channel, while the fluorescence in the FRET channel stayed at the same level. It can be partly explained by the overlaying fluorescence spectra for donor and acceptor, therefore the rise of acceptor fluorescence was partly compensated by the drop of donor fluorescence. This also means that for some applications, the indicator can be used as intensiometric, with only the donor channel used for Ca^2+^ imaging.

Although we have not directly compared a signal-to-noise ratio of iGECInano and indicators of GCaMPs series, we assume that in cell cultures, iGECInano or any of the currently available NIR GECIs would have a lower signal-to-noise ratio compared to GCaMP6, as the latter has a higher molecular brightness (123 for GCaMP6 vs. 12–37 for NIR GECIs) and a much higher dynamic range (60x for GCaMP6 vs. 4-8x for NIR GECIs) (Table 1). However, deep-tissue imaging GFP-based indicators, such as GCaMP6, will suffer from signal loss from absorbance by body pigments and light-scattering and higher background from autofluorescence. Emerging NIR GECIs can help overcome this problem and, as they evolve, have the potential to compete with golden standard GFP-based indicators in the future.

## Conclusion

iGECInano, developed in this article, has sufficient brightness and photostability in cells, fast response kinetics, and low molecular size, therefore, combining the advantages of the current state-of-the-art ratiometric iGECI and intensiometric NIR-GECO2G.

Further improvement of iGECI could consist of additional reduction of its size by using a smaller FRET-acceptor, such as red-shifted NIR FP designed from cyanobacteriochrome, which is not currently available yet. Also, a small, single-domain miRFP670nano is amenable to circular permutations as its N- and C-termini are located closely. The circularly permuted miRFP670nano as a FRET-donor could further improve the FRET changes between calcium-free and calcium-loaded states.

iGECInano could be further used in spectral crosstalk-free combinations with popular GFP-based biosensors, allowing the monitoring of several cellular parameters, and with optogenetic actuators for simultaneous optical modulation and monitoring of calcium dynamics in all-optical setups. Moreover, appending a soma-targeting sequence to iGECInano and creating a transgenic mouse stably expressing iGECInano for efficient incorporation of available endogenous biliverdin, as well as the combination of this genotype with biliverdin reductase-A knock-out, should increase the signal-to-noise ratio, allowing advanced *in vivo* applications.

## Materials and Methods

### Design of Bacterial and Mammalian Plasmids

miRFP670nano and a truncated version of miRFP720 (deletion of 17 N-terminal amino acids, miRFP720Δ17N) were PCR-amplified from pmiRFP670nano and pmiRFP720-N1 plasmids. A minimal TnC (TnC_m_) sensing module was PCR-amplified from Twitch-2B-pcDNA3 (Addgene #49531). A NIR-GECO2G gene was PCR-amplified from pAAV-CAG-NIR-GECO2G (Addgene #159605). A plasmid pUCmini-iCAP-PHP.eB encoding-modified AAV2/9 capsid was kindly provided by Prof. V. Gradinaru (California Institute of Technology, Addgene #103005). A pHelper plasmid was from the AAV-Helper Free System kit (Agilent #240071). Plasmids encoding iGECI were previously constructed in our lab. For the cytoplasm-restricted localization, nuclear export (NES) signal peptide NELALKLAGLDINK was appended to the N-terminus of iGECInano *via* a short GGGS linker using a primer-extension PCR.

For the bacterial expression of GECI variants, pBAD/HisD vector (Life Technologies/Invitrogen) was used. Mammalian expression plasmids were based on a pEGFP-N1 vector (Clontech) with a standard CMV promoter. For the expression in dissociated neurons, iGECInano or NIR-GECO2G genes were cloned into pAAV-CAG-mRuby (Addgene #99123) in place of mRuby.

### Molecular Evolution of iGECInano

DNA fragments encoding miRFP670nano, TnC_m_, and miRFP720Δ17N were PCR amplified and ligated into the pEGFP-N1 vector. A set of constructs was created with miRFP670nano3 at the N-terminus and miRFP720 at the C-terminus and vice versa connected directly or with 1–4 a.a. randomized linkers. All variants were cloned into the pBAD/His-D vector and transformed into BL21 AI *E. coli* host (ThermoFisher Scientific) containing a pWA23h plasmid encoding heme oxygenase (HO) for BV synthesis. Cells were grown overnight in an LB medium containing 0.02% rhamnose and 0.05% arabinose for induction of HO and iGECI syntheses, respectively.

The library of clones was sorted with FACS, using double-positive gating for eliminating non-fluorescent clones resulting from stop-codons and frameshifts. The pre-sorted library was plated on Petri dishes containing 0.02% rhamnose and 0.05% arabinose so that approximately 100 colonies were growing on each dish. Dishes were incubated overnight at 37°C, then for 24 h at 18°C. Colonies were transferred to nitrocellulose membranes and permeabilized by spraying with Ca^2+^-free solution (30 mM MOPS, pH 7.5, 100 mM KCl, 50 μg/ml poly-L-lysine, 50 μg/ml ionomycin). Membranes were incubated for 5 min, and basal fluorescence in the donor (ex. 605 nm, em. 680 nm) and FRET (ex. 605 nm, em. 720 nm) channels were acquired using an IVIS instrument (Perkin Elmer/Caliper Life Sciences). Then, membranes were treated by spraying with high Ca^2+^ solution (30 mM MOPS, pH 7.5, 100 mM KCl, 50 μg/ml poly-L-lysine, 50 μg/ml ionomycin, 100 mM CaCl_2_), incubated for 5 min, and the Ca^2+^-loaded state of fluorescence was recorded using the same filter sets. Data were analyzed using ImageJ software. 12–16 dishes or approximately 1,200–1,600 colonies were screened on membranes for each library per a round of screening.

Clones with the best Ca^2+^-loaded/basal fluorescence ratio (typically 50–100 colonies per a round of screening) were transferred to 5 ml LB liquid culture in 24 deep-well plates containing 0.02% rhamnose and incubated for 2 h at 37°C on a rotating shaker. Then, arabinose was added to 0.05%, the culture was incubated for 3 h at 37°C, then for 24 h at 25°C. The bacterial pellets were lysed with B-PER (ThermoFisher Scientific). The cleared lysates were transferred to 96-well plates and divided. One part was loaded with 1 mM CaCl_2_ and another one with 2 mM EGTA. Fluorescences of the donor (ex. 605 nm, em. 670 nm) and FRET (ex. 605 nm, em. 720 nm) were acquired using the SpectraMax M2 plate reader (Molecular Devices).

The best performing constructs were subcloned into a mammalian expression vector and evaluated in HeLa cell lysates. HeLa cells were transiently transfected using FuGENE HD (Promega); 24 h after transfection, 5 µM BV was added. 48 h after transfection, the cells were harvested and lysed with M-PER (ThermoFisher Scientific). Lysates were cleared by centrifugation and divided in two samples; one part was loaded with 1 mM CaCl_2_ and another one with 2 mM EGTA. Fluorescence spectra were recorded with the FluoroMax-3 spectrofluorometer (Horiba). The best performing clones were subjected to a new round of L1 and L2 evolutions in *E. coli* and HeLa cells. Three rounds of evolution and screening were performed to identify the final indicator variants.

### Protein Purification and *In Vitro* Characterization

The iGECI constructs with polyhistidine tags at the N-terminus were expressed in the BL21-AI host (Life Technologies/Invitrogen) containing a pWA23h plasmid. Bacteria were grown in an LB medium supplemented with ampicillin, kanamycin, and 0.02% rhamnose for 2 h at 37°C, followed by the induction of protein expression with 0.05% arabinose for 3 h at 37°C, then 24 h at 25°C on a rotating shaker. The proteins were purified using Ni-NTA agarose (Qiagen).

For absorbance measurements, a Hitachi U-2000 spectrophotometer was used. Fluorescence spectra in the range of 650–740 nm were recorded with the FluoroMax-3 spectrofluorometer.

Ca^2+^ titrations were carried out using EGTA-buffered Ca^2+^ solutions (Calcium Calibration Buffer Kit, Life Technologies). We prepared buffers by mixing a Ca^2+^-EGTA buffer and an EGTA buffer to give free Ca^2+^ concentrations ranging from 0 to 39 μM at 25°C. Fluorescence intensities were plotted against Ca^2+^ concentrations and fitted by a sigmoidal binding function to determine Kd.

Using a series of Hydrion buffers (Micro Essential Laboratory), pH stability was studied in the presence of either 2 mM EGTA or 1 mM CaCl_2_. Fluorescence was excited at 620 nm, and the emission was recorded at 640–760 nm. The area under the spectra at different pH values was quantified.

A comparison of the brightness of iGECInano, iGECI, and NIR-GECO2G was performed in HeLa cells transiently transfected with FuGENE HD. For a BV-saturated condition, 5 µM BV was added 24 h after transfection. 48 h after transfection, cells were analyzed with a BD LSRII flow cytometer using a 640 nm excitation laser and a 647 nm long-pass edge emission filter. The NIR fluorescence intensity was normalized to the efficiency of absorption at 640 nm for each indicator. Flow cytometry gating was performed using intact, single cells.

Photobleaching measurements of the indicators in live HeLa cells and dissociated mouse neurons were performed with the ×100, 1.4 NA oil-immersion objective lens (UPlanSApo, Olympus) and a 605/30-nm excitation and a 647 nm long-pass emission filters at a light power density of 14 mW cm^−2^ measured at the back aperture of the objective lens (∼8.3 W cm^−2^ at the specimen plane) and normalized to the efficiency of absorption at 605 nm for each indicator.

### Histamine Oscillations

To measure Ca^2+^ transitions evoked by histamine, HeLa cells were transiently transfected with iGECI using FuGENE HD (Promega) and cultured for 48 h. Then, the medium was changed to Live Cell Imaging Solution (Life Technologies, Invitrogen), supplemented with 1 mM CaCl_2_, 100 mM KCl and 1 mM D-glucose, and basal fluorescence in the donor (excitation, 605 nm; emission, 667/30 nm) and FRET (excitation, 605 nm; emission, 725/40 nm) channels was recorded. Time-lapse imaging was performed with an Olympus IX81 inverted epifluorescence microscope, equipped with a 200 W xenon lamp (Sutter Instruments) and a ×60, 1.35 NA oil-immersion objective lens (UPlanSApo, Olympus). The microscope was operated with SlideBook v. 6.0.8 software (Intelligent Imaging Innovations). A histamine solution was added to the cells to a final concentration of 100 μM, and the fluorescence was recorded for 10 min. After that, the imaging solution with Ca^2+^ and histamine was replaced by a Ca^2+^-free imaging solution, and the cells were incubated for 5 min to equilibrate Ca^2+^. Then, an imaging solution containing 2 mM EGTA was added to the cells, and fluorescence was recorded for another 5 min. The images were analyzed using ImageJ software.

### Preparation of High-Titer AAVs

AAV particles were obtained as described (Challis et al., 2019). Briefly, plasmid DNA for AAV production was purified with the NucleoBond Xtra Maxi EF kit (Macherey-Nagel), and AAV-293T cells (Agilent) were co-transfected with pAAV-CAG-NIR-GECO2G or pAAV-CAG-iGECInano plasmid, AAV capsid plasmid pUCmini-iCAP-PHP.eB, and pHelper using polyethyleneimine (PEI; Santa Cruz). Cell medium was collected 72 h after transfection. The cells and the medium were collected 120 h after transfection and combined with the medium collected at 72 h. The cells were harvested by centrifugation and then lysed with a salt-active nuclease (HL-SAN, ArcticZymes). Polyethylene glycol (PEG, 8%) was added to the medium, and the mixture was incubated for 2 h on ice and then pelleted. The PEG pellet was treated with HL-SAN and combined with the lysed cells. The cell suspension was clarified by centrifugation. The supernatant was applied on an iodixanol gradient and subjected to ultracentrifugation for 2  h and 25 min at 350,000 g. The virus fraction was collected, washed, and enriched at an Amicon Ultra-15 100,000 MWCO centrifuge device. The virus titer was determined by qPCR. An aliquot of the virus was treated with DNase I and proteinase K and then used as a template for qPCR. A pAAV-CaMKII–iGECInano plasmid of known concentration that was digested with EcoRI was used as a reference.

### Imaging of Primary Dissociated Neurons

Neurons were isolated from the hippocampi of P0–P1 Swiss Webster mice using a published protocol (Beaudoin et al., 2012) and cultured in a Neurobasal Plus medium with B-27 Plus Supplement (Gibco), additional GlutaMAX (1 mM; Gibco), 100 U ml^−1^ penicillin, and 100 μg ml^−1^ streptomycin, on poly (D-lysine) (EMD Millipore)-coated glass coverslips (thickness, 0.13–0.17  mm; diameter, 12 mm; ThermoFisher Scientific) at a density of ∼70,000 cells per coverslip. Half of the medium was exchanged twice per week. For experiments with AAVs, neurons were transduced at DIV7 with 10^9^ viral genomes (vg) per well (in 24-well plates) and recorded at DIV16–DIV18 at 37°C. The Grass S48 stimulator (Grass Instruments) and custom platinum electrodes (0.5-mm diameter) were used for field stimulation (1 ms single pulses, 50 V). The following synaptic transmission inhibitors were applied: 10 μM CNQX (R&D Systems), 10 μM gabazine (Santa Cruz Biotechnology), 10 μM (R)-CPP (Enzo Life Sciences), and 1 μM (S)-MCPG (Cayman Chemicals). A field stimulation using 1 ms single pulses, 50 V, in the presence of synaptic transmission inhibitors was previously described to generate single APs in cultured neurons (Wardill et al., 2013). A 617 nm light-emitting diode (LED) (Mightex Systems) was used for fluorescence excitation. The excitation filter was 620/15 nm, with a 640LP dichroic mirror, and the emission filter was 667/30 nm for iGECI and iGECInano and 720/40 nm for NIR-GECO2G. The frame rate was 10 Hz for NIR-GECO2G and 5 Hz for iGECI and iGECInano. Fluorescence was recorded using an Orca-Flash4.0 LT camera (Hamamatsu), an Olympus IX81 microscope, and a LUCPlanFLN ×20, 0.45 NA air objective lens (Olympus). Light power density at the specimen plane was 1.4 W cm^−2^ (six-folds lower than in the photobleaching experiments), and the total duration of imaging was less than 0.5 h. The bath solution contained (in mM) 125 NaCl, 2.5 KCl, 1 MgCl_2_, 10 HEPES, 3 CaCl_2_, and 30 D-glucose at pH 7.3, 305–307 mOsm. The stimulator, camera, and LEDs were controlled by Master-8 (AMPI) and MatLab R2018b (MathWorks). Each neuronal recording was performed from an individual coverslip. Stimulation evoked changes in the fluorescence were quantified relative to the 10 s baseline before stimulation. Time to the peak was measured as the time between stimulus onset and the peak of the evoked response. Decay time was quantified as the time constant of a fitted single exponential curve. Data analysis was performed using Igor Pro 9 software. A statistical analysis was performed using the Kruskal–Wallis test correcting for the False Discovery Rate. We inform *p* values (q) corrected for multiple comparisons. We used GraphPad Prism v. 9.3.1 software.

## Data Availability

The original contributions presented in the study are included in the article; further inquiries can be directed to the corresponding author.
